# Epigenetic rejuvenation

**DOI:** 10.1111/j.1365-2443.2012.01595.x

**Published:** 2012-05

**Authors:** Maria Manukyan, Prim B Singh

**Affiliations:** 1 Albert-Ludwigs-Universität Freiburg, BIOSS Centre for Biological Signalling Studies, Zentrum für Biosystemanalyse – ZBSAHabsburgerstrasse 49, 79104 Freiburg, Germany; 2 Fächereverbund Anatomie, Institut für Zell and Neurobiologie, Charite – Universitätsmedizin10117 Berlin, Germany

## Abstract

Induced pluripotent stem (iPS) cells have provided a rational means of obtaining histo-compatible tissues for ‘patient-specific’ regenerative therapies (Hanna *et al.* 2010; Yamanaka & Blau 2010). Despite the obvious potential of iPS cell-based therapies, there are certain problems that must be overcome before these therapies can become safe and routine (Ohi *et al.* 2011; Pera 2011). As an alternative, we have recently explored the possibility of using ‘epigenetic rejuvenation’, where the specialized functions of an old cell are rejuvenated in the absence of any change in its differentiated state (Singh & Zacouto 2010). The mechanism(s) that underpin ‘epigenetic rejuvenation’ are unknown and here we discuss model systems, using key epigenetic modifiers, which might shed light on the processes involved. Epigenetic rejuvenation has advantages over iPS cell techniques that are currently being pursued. First, the genetic and epigenetic abnormalities that arise through the cycle of dedifferentiation of somatic cells to iPS cells followed by redifferentiation of iPS cells into the desired cell type are avoided (Gore *et al.* 2011; Hussein *et al.* 2011; Pera 2011): epigenetic rejuvenation does not require passage through the de-/redifferentiation cycle. Second, because the aim of epigenetic rejuvenation is to ensure that the differentiated cell type retains its specialized function it makes redundant the question of transcriptional memory that is inimical to iPS cell-based therapies (Ohi *et al.* 2011). Third, to produce unrelated cell types using the iPS technology takes a long time, around three weeks, whereas epigenetic rejuvenation of old cells will take only a matter of days. Epigenetic rejuvenation provides the most safe, rapid and cheap route to successful regenerative medicine.

## Introduction

Somatic cell nuclear transfer (SCNT; animal cloning) has shown that a newborn clone can be derived from an old, differentiated cell (Wilmut *et al.* 1997). Likewise, old cells can be returned back to a pluripotent ES-cell-like state (iPS cells) using ‘reprogramming factors’, Oct4, KLf4, Sox2 and c-Myc; iPS cells can thereafter be redifferentiated back to specialized cell types from which they were derived (Yamanaka & Blau 2010). For both methods, the process of ‘rejuvenation’ involves passage through an ES-cell-like stage and the erasure of an ‘aged’ epigenotype to be replaced by a ‘youthful’ one. A key question is whether ‘developmental reprogramming’ to the embryonic stage can be molecularly separated from ‘age reprogramming’ whereby a cell is simply returned back to a more ‘youthful’ state. Reprogramming the age of a cell in isolation while maintaining its differentiated state, thus effectively rejuvenating the specialized functions peculiar to that cell type, is termed ‘epigenetic rejuvenation’. Should epigenetic rejuvenation be achieved it would have profound consequences for Medicine and the relief of human suffering. In this article, we explore furthermore the goal of epigenetic rejuvenation and discuss model systems, using key epigenetic components, which could provide insight into the molecular mechanisms involved.

## Ageing, cellular senescence, and the heterochromatin connection

A variety of evolutionary theories, including the ‘mutational accumulation’ (Medawar 1952), ‘antagonistic pleiotropy’ (Williams 1957), and the ‘disposable soma’ (Kirkwood 1977) theories, have been advanced to explain the aging of, and the range of life span found in, Metazoan species. Although these evolutionary considerations have provided a backdrop in terms of how ageing might have evolved, we must look to the molecular and cellular mechanisms that underpin age-related deterioration for possible regimes that could enhance healthy aging. At the molecular level, the most well-studied mechanisms that result in age-related deterioration of cellular function involve damage to macromolecules. They include changes in chromatin structure (Oberdoerffer & Sinclair 2007), oxidative damage to DNA and proteins (Holzenberger *et al.* 2003), telomere attrition (Kim *et al.* 2002), and the deterioration of the cellular repair machinery (Sedelnikova *et al.* 2004). The damage-induced deterioration of cellular function is considered to effect changes in cellular phenotype, which we observe as ageing. And one of the most well-studied phenotypic effects of ageing is cellular senescence (Campisi & d’Adda di Fagagna 2007). Notably, a recent synthesis has concluded that different intrinsic and extrinsic stressors can result in the damage of macromolecules during ageing and this damage can, in turn, regulate signaling pathways that drive cells toward senescence (Adams 2009).

Cellular senescence was first documented as an *in vitro* phenomenon around 50 years ago and described as the irreversible growth arrest of all cells in a culture that is triggered by the exhaustion of their cell division potential (Hayflick & Moorhead 1961). Such replicative senescence is rarely reached *in vivo* (Kreiling *et al.* 2011). Nevertheless, senescent cells can be found in aged tissues taken from a variety of species, including mice, baboons and humans (Herbig *et al.* 2006; Jeyapalan *et al.* 2007; Wang *et al.* 2009). Tissues taken from these aged animals are, in fact, a mixture of senescent cells interspersed with normal cells (old, perhaps age-compromised cells but not yet senescent) (Bahar *et al.* 2006; Herbig *et al.* 2006). As explained earlier, senescence can be triggered by several stressors that result in molecular damage and not just by exhaustion of replicative potential (Adams 2009). Thus, different tissues that may have been exposed to different levels of the stressors through life – including pathology – are likely to have different numbers of senescent cells in them.

A key finding in recent years has come from *in vitro* studies, which have shown that cellular senescence, in response to replicative exhaustion or external stressors (specifically, oncogene-induced cellular senescence), is associated with dramatic nuclear reorganization and formation of so-called senescent-associated heterochromatin foci (SAHF) (Narita *et al.* 2003). SAHF contain within them a variety of markers the most notable being the repressive me3K9H3 determinant of the histone code, heterochromatin protein 1 (HP1) proteins, and the variant histone macro H2A (mH2A) (Narita *et al.* 2003; Zhang *et al.* 2005). SAHF are considered to sequester proliferation-promoting genes (E2F-responsive genes) and thereby trigger the senescent state of cells (Narita *et al.* 2003; Zhang *et al.* 2005). The strong correlation between SAHF and senescence has led workers to explore the association of SAHF and senescence *in vivo* (Kreiling *et al.* 2011). The data have indicated that, *in vivo*, SAHF formation in senescent cells is not common place and that even *in vitro* the frequency of SAHF formation can vary greatly depending on cell type (Kreiling *et al.* 2011; Di Micco *et al.* 2011; Kosar *et al.* 2011). However, one striking outcome of these studies is that there is a significant, quantitative, passage-associated increase in two heterochromatin markers, HP1β and mH2A, in the nuclei of cells approaching senescence *in vitro* and, for mH2A, a tight correlation with ageing of cells *in vivo* (Kreiling *et al.* 2011).

HP1β and mH2A are known to be involved in the epigenetic regulation of gene expression (Billur *et al.* 2010; Muthurajan *et al.* 2011). HP1β binds to the histone modification me3K9H3 including SAHF and is, in addition, involved in telomere physiology, assembly at DNA repair foci and the regulation of the Oct4 pluripotency factor; these latter functions have been highlighted as being useful for the dissection of ‘epigenetic rejuvenation’ (Singh & Zacouto 2010). mH2A is considered to be involved in the epigenetic facultative heterochromatinization of the inactive X-chromosome in female cells (Chow & Brown 2003). Notably, HP1β and mH2A co-purify with the same chromatin fragments (Changolkar & Pehrson 2006) and, as explained, they both co-localize in SAHF (Narita *et al.* 2003; Zhang *et al.* 2005). These data indicate that key epigenetic modifiers, including HP1β and mH2A, are likely to be involved in regulating exit from the cell cycle in response to stressors that drive cellular senescence and thereby contribute to the ageing of an organism.

## Ageing as an epigenetic phenomenon: epigenetic rejuvenation

**Figure 1:**
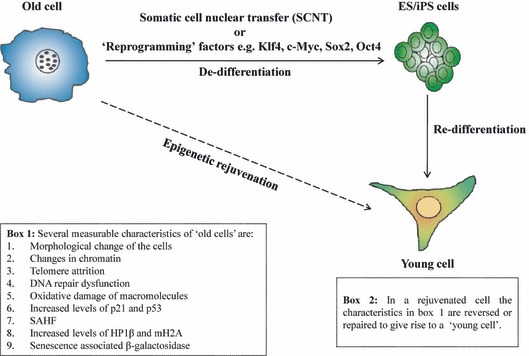
Epigenetic rejuvenation bypasses the ES/iPS cell stage. Box 1 describes the characteristics of an old senescent cell. Senescent cells possess chromatin damage, telomere attrition, morphological changes in shape, dysfunction in DNA repair mechanisms, oxidative damage, increased levels of the cell cycle inhibitors p21 and p53, increased levels of the epigenetic modifiers heterochromatin protein 1 (HP1β) and mH2A (variant histone macro H2A), presence of SAHF (senescent-associated heterochromatin foci) (depicted as large ‘dots’ in the old cell), and expression of β-galactosidase activity. The horizontal arrow depicts the dedifferentiation pathway from the senescent cell to the ES/iPS cell stage after somatic cell nuclear transfer (SCNT) or introduction of the ‘reprogramming factors’. Epigenetic rejuvenation of old cells (diagonal arrow) can be achieved by ‘conditioning’ of old cells within the oocyte cytoplasm via SCNT as explained in the text. For simplicity, we have not shown somatic cell transfer into the germinal vesicle (GV) of frog oocytes but GV system does represent another model system for the study of ‘epigenetic rejuvenation’, as detailed in the text. The vertical arrow downwards depicts the redifferentiation of the ES/iPS cells into a young differentiated cell type, which has reversed or repaired all the senescence characteristics of the old cell (see box 2). The young cell has lost its SAHF.

Evidence that ageing is likely to have a significant epigenetic component comes from animal cloning experiments using SCNT. SCNT was first described in amphibians (Briggs & King 1952; Elsdale *et al.* 1960) and much later in mammals (Wilmut *et al.* 1997). These influential experiments showed that the differentiated state was reversible and not accompanied by irreversible gene loss, excepting for particular lineages like B and T cells; differentiation was the result of epigenetic regulation of gene expression. Thus, nuclear reprogramming of somatic cells was seen as a process where the developmental potential of the somatic cell was reset back to the totipotent, embryonic state from which the whole of development could be recapitulated. The result was a newborn clone, which was genetically identical to the somatic cell transferred into the recipient oocyte. In all respects, the clone was normal including the possession of a normal lifespan, even when the somatic cell was derived from an old donor (Mizutani *et al.* 2008; Wakayama *et al.* 2010). Thus, the age-associated macromolecular damage found in ageing cells can be reversed: SCNT shows that mechanism(s) exist to rejuvenate cells (Fig. 1; boxes 1 and 2). It also follows that typical senescence-associated increase in two of the heterochromatin markers, HP1β, and mH2A (Kreiling *et al.* 2011) will be reversed. More recently, the seminal studies of Yamanaka and colleagues have shown that ‘reprogramming factors’ can reprogram somatic cells into iPS cells, even from an elderly, 82-year-old donor (Dimos *et al.* 2008). In an important extension to the iPS cell work, iPS cells derived from mouse fibroblasts could, *via* tetra-ploid complementation give rise to newborn clones, as seen after SCNT (Kang *et al.* 2011). It would seem that induction of iPS cells can also reset the ageing clock.

The observation that the age program could be reset after SCNT and iPS cell induction, led to the concept that mechanisms exist to rejuvenate somatic cells (Surani & McLaren 2006). To explore the phenomenon of rejuvenation *via* SCNT and by iPS cell induction furthermore, it will be necessary to dissect the reprogramming process(es). At face value, nuclear reprogramming during SCNT and iPS cell induction appears a seamless process: ‘developmental reprogramming’ to the totipotent state is concomitant with ‘age reprogramming’ where the age of the donor somatic nucleus is wiped clean. But the question remains: can developmental reprogramming be disentangled from age reprogramming? If so, then the differentiated, specialized functions of a cell could be left unchanged, whereas the age of the cell could be reprogrammed. In short, a differentiated cell could be rejuvenated, simply made younger without affecting its specialized function(s) that are peculiar to its differentiated state (Fig. 1). We have called this process of age reprogramming ‘epigenetic rejuvenation’ (Singh & Zacouto 2010).

## Model systems for age reprogramming studies

The current interest in the process of nuclear reprogramming during iPS cell induction and after SCNT has enabled rapid progress in describing the kinetics and stability of the reprogramming and the factors that restrict efficient reprogramming (Nagy & Nagy 2010; Jullien *et al.* 2011). These studies provide a sound foundation for investigations into whether age reprogramming (‘epigenetic rejuvenation’) can be separated from developmental reprogramming.

**Figure 2:**
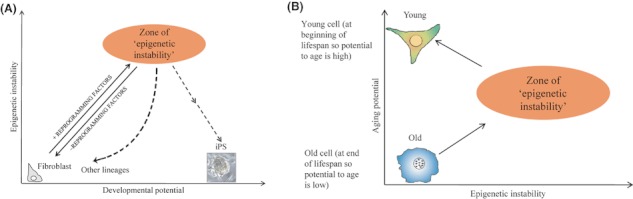
iPS system for the study of epigenetic rejuvenation. In (A) is shown the relationship of epigenetic instability (the *y*-axis) after introduction of the ‘reprogramming factors’ to the developmental potential of cells (the *x*-axis). After introduction of the reprogramming factors, cells set out on the path toward iPS cells and pass, after 4–7 days a zone of ‘epigenetic instability’ where the fate of the cells undergoing reprogramming is pliant. Using defined media, the ‘unstable’ cells can be forced down different developmental pathways giving rise to other cell linages (Efe *et al.* 2011). Of interest is the fact that if the expression of the reprogramming factors is silenced in the ‘zone of epigenetic instability’, the cells return back to being fibroblasts (Nagy & Nagy 2010). In (B), reprogramming factors have been introduced into an old senescent fibroblast (with SAHF represented by ‘dots’ in the nucleus) that has a low potential to age (*y*-axis) as it is already old. It then sets out on the path toward becoming an iPS cell. As it does so its epigenetic instability increases (*x*-axis) and passes through the ‘zone of epigenetic instability’. On reaching the ‘zone of epigenetic instability’, reprogramming factor expression is silenced and the cells return back to the fibroblast ‘ground-state’. In the diagram given, the fibroblast produced after passage through the ‘zone of epigenetic instability’ is a young fibroblast (with no SAHF) with a high potential to age. This is to be tested.

A recent observation that is likely to be important for the study of epigenetic rejuvenation comes from work that has investigated the kinetics and stability of iPS reprogramming. It has been observed that expression of reprogramming factors in mouse embryonic fibroblasts (MEFs) for 4–7 days destabilizes the MEF epigenotype without committing the cells to an iPS cell fate (Nagy & Nagy 2010; Efe *et al.* 2011). This destabilization is not enough to ensure that cells follow the iPS trajectory because removal of the reprogramming factors, whereas cells are within this ‘window’ of instability results in their return back to MEF ‘ground-state’ (Nagy & Nagy 2010). An interesting extension of this study has taken advantage of the transient instability of the MEF epigenome after introduction of the reprogramming factors to circumvent passage through the pluripotent iPS cell stage and thereby directly reprogram fibroblasts into cardiomyocytes (Efe *et al.* 2011). These observations beg the question of whether the return trip through the transient, epigenetically unstable state affects (resets) the age program in isolation; the developmental program remains unchanged because the cells gravitate back to the ‘ground-state’ upon removal of the ‘reprogramming factors’ (Fig. 2a,b). Our current work is directed toward testing this hypothesis using senescent primary human fibroblasts expressing either HP1β- or mH2A-GFP from their endogenous promoters. As shown in Fig. 2b, it is possible to test – using quantitative measurement of fluorescence intensity over individual nuclei – whether the elevated levels of HP1β- or mH2A-GFP (that may be contained within SAHF) return to their lower, ‘youthful’ levels after the round-trip to the epigenetically unstable zone and back to the fibroblast ‘ground-state’ again. Measurement of HP1β- and mH2A-GFP mobility by FRAP in senescent human fibroblasts (particularly within any SAHF) during the return journey may provide a quantitative measure of age reprogramming and thus an assay for chemicals and media that can facilitate the age reprogramming process.

Progress in defining the factors that restrict reprogramming efficiency has come from the study of reprogramming of mammalian cells after their incubation in the germinal vesicle (GV) of the frog oocyte (Halley-Stott *et al.* 2010). Recent work using the GV frog oocyte system has shown that one of the factors that restrict reprogramming efficiency is the variant histone mH2A (Pasque *et al.* 2011). This is the same mH2A described previously whose levels increase in a passage-dependent manner as cells approach senescence and later can be found as a constituent of SAHF (Kreiling *et al.* 2011). These data indicate that age reprogramming may need to overcome chromatin structures that are refractory to reprogramming. Efficient age reprogramming may require knock-down’ (KD) of specific chromatin components, such as HP1β and mH2A, before SCNT (Fig. 1) and iPS cell induction (Fig. 2). To that end, our recent work has shown that siRNA KD of HP1β mRNA can trigger cell division in senescent human diploid fibroblasts *in vitro* (J.P. Brown and P.B. Singh, unpublished).

A key feature of the GV oocyte is that it represents an environment where direct reprogramming of gene expression can take place without cell division and without any new cell types being generated (Jullien *et al.* 2011). As such, the GV oocyte represents a ‘laboratory’ in which the study of reprogramming can be undertaken without overt dedifferentiation. It provides a model system for the study of epigenetic rejuvenation. It is unclear at present whether epigenetic rejuvenation of old nuclei after SCNT occurs soon after introduction of the nucleus into the oocyte itself or requires that the old nucleus undergoes several rounds of cell division after embryo reconstruction. If epigenetic rejuvenation occurs early after SCNT, in the oocyte itself, then the partial cloning technique (Singh & Zacouto 2010) could be used where permeabilized senescent cells can be temporarily ‘parked’ in the GV of the frog oocyte and then, after re-sealing, put back into culture to test whether exposure to the GV milieu results in loss of any of the known senescent cell characteristics (Fig. 1; boxes 1 and 2). Similar experiments can be undertaken with mammalian oocytes where permeabilized senescent cells are injected into metaphase II arrested oocytes and after temporary conditioning in the oocyte cytoplasm for varying lengths of time the permeabilized cells are returned to tissue culture to investigate whether there is any loss of the senescent characteristics within the nuclei of the donor cell (Fig. 1; boxes 1 and 2). We are mindful that the GV of the frog oocyte and the metaphase II arrested mammalian oocyte represent environments that are inimical to cell division, thus the resumption of cell division may not be a good read-out of epigenetic rejuvenation of senescent cells after conditioning in GV and metaphase II arrested oocytes. In this context, the partial cloning technique (Singh & Zacouto 2010) may be better suited to transient conditioning of permeabilized senescent cells in preactivated mammalian oocytes.

If rejuvenation of senescent nuclei after SCNT requires a few cell divisions, such that the senescent nuclei need to replicate as the embryo develops, the experimental regimes are likely to be more complicated. For example, nuclei from senescent fibroblasts could be transferred (electro-fused or microinjected) into MII arrested oocytes that are subsequently artificially activated to develop. The haploid maternal chromosome complement is then removed at the first interphase and furthermore development takes place under control of the diploid senescent cell genome that is being reprogrammed; retention of the haploid maternal chromosome complement for as long as possible, until the first interphase, may aid ‘age reprogramming’ (Noggle *et al.* 2011). Reprogramming then proceeds through the 2, 4, 8 to the 32-cell/morula stage. At each of these stages, diploid descendants of the transferred senescent nucleus are then transplanted into recipient cytoplasts (Pralong & Verma 2006) derived from senescent fibroblasts. The reconstructed cells can then be analyzed for loss of the senescent characteristics of the donor nuclei (Fig. 1; boxes 1 and 2) and whether the ‘age reprogrammed’ nuclei can trigger re-entry of the reconstructed cells into the cell cycle and sustain cell division.

## Perspectives

Work on SCNT and iPS cells has revealed that age reprogramming –‘epigenetic rejuvenation’– is possible. The key question is whether age reprogramming can be disentangled from developmental reprogramming. Should this be achievable the consequences for Medicine would be profound. It would avoid the need to artificially pass through an embryonic stage – either by nuclear transfer or by the iPS method – to rejuvenate cells. One would simply be able to take aged cells from a patient and then return back to the patient their own, histocompatible, rejuvenated heart cells, liver cells *etc.* The need for model systems for the study of ‘epigenetic rejuvenation’ is paramount. Recent work on the kinetics and stability of the reprogramming process and the factors that cause resistance to reprogramming now provide a framework for understanding the mechanism(s) that underpin ‘epigenetic rejuvenation’. In particular, we suggest that the chromatin structures that resist the reprogramming process are likely to be important. And as a route into investigating these mechanisms, we have described experiments, currently underway, involving measuring the mobility and manipulating the levels of two key epigenetic modifier proteins, HP1β and mH2A, which could provide insight into ‘epigenetic rejuvenation’.

## Note added in proof

During the review process, a new paper by Lapasset *et al.*, ‘Rejuvenating senescent and centenarian human cells by reprogramming through the pluripotent state’ (*Genes and Development* (2011) **25**, 2248–2253) was published. These workers showed that 1 week after introduction of the 6 ‘pluripotency’ factors into SAHF-containing senescent fibroblasts that there was loss of SAHF and proliferation was resumed after 18–20 days. Other parameters of rejuvenation (e.g., telomere length) were not assayed during this crucial period, where we suspect epigenetic rejuvenation is taking place. Instead, these workers allowed reprogramming to pass through the iPS cell stage and then onto redifferentiation before measuring the rejuvenated physiology of the differentiated cells.
